# Save Our Roads from GNSS Jamming: A Crowdsource Framework for Threat Evaluation

**DOI:** 10.3390/s21144840

**Published:** 2021-07-15

**Authors:** Roi Yozevitch, Revital Marbel, Nir Flysher, Boaz Ben-Moshe

**Affiliations:** 1 Department of Computer Science, Holon Institute of Technology, Holon 58102, Israel; 2 Department of Computer Science, Ariel University, Ariel 40700, Israel; revi85@gmail.com (R.M.); nirflysher@gmail.com (N.F.); benmo@g.ariel.ac.il (B.B.-M.)

**Keywords:** global orientation sensor, accurate orientation for autonomous robotics

## Abstract

Global Navigation Satellite Systems (GNSS) jamming is an acute problem in the world of modern navigation. As more and more applications rely on GNSS for both position and timing, jamming ramifications are becoming more severe. In this paper we suggest a novel framework to cope with these threats. First, a Bayesian jamming detection algorithm is introduced. The algorithm can both detect and track several jammers in a pre-defined region of interest. Then, a jamming coverage map algorithm is offered. Similar to cellular 3G/4G coverage maps, such a map can detect “weak” GNSS reception spots and handle them. Since jamming interference can be a dynamic phenomenon (e.g., a vehicle equipped with a jammer), the coverage map changes with time. Thus, interference patterns can be detected more easily. Utilizing the offered algorithm, both on simulation and field experiments, we have succeeded to localize an arbitrary jammer(s) within the region of interest. Thus, the results validate the viability of the proposed method.

## 1. Introduction

It is almost impossible to imagine modern life without the use of GNSS. From automobile and drone navigation, through assets tracking and to accurate synchronization for cellular base stations. Moreover, some of these projects are also becoming autonomous. Although self driving vehicles heavily rely on other sensors, such as vision and LIght, Detection And Ranging (LIDAR), to navigate they also rely on GNSS receivers to obtain a coarse position in the environment [1].

There are two sides to this dependency. As we increasingly rely on these systems, the ramifications of sabotaging them also grows. In fact, it is well known that these signals are extremely susceptible to radio frequency (RF) noise and other kinds of threats. An evaluation of the impact of Jamming Signals on GNSS receivers is described here [2]. A foe that blocks or manipulates GNSS signals can cause a real-life threat [3].

Previous GNSS attacks that resulted in loss of position fix, speed inputs, and collision avoidance capabilities have raised alerts. The famous US-North Korea radio frequency “war” in which North Korean jammed global positioning system (GPS) signals in South Korea three times over two years underscored the system’s potential vulnerability. Waterman [4] is a good example of these dangers. Not all attacks are intentional, however. In one case, an eBay-purchased GNSS jammer (that cost less than 50 USD) almost caused an unfortunate accident [5]. It is important to mention that jamming attacks also affect critical infrastructure such as airports and naval ports, as elaborated in [6].

Another kind of threat can be achieved by GNSS clock drifting. Because GNSS satellites are extremely accurate (each satellite is equipped with four atomic clocks), cellular backhauls rely on them to synchronize their base station clocks. Such attacks can paralyze a cellular base station completely [7].

In the basic naive form of attack, called GNSS jamming, RF noise is transmitted in the carried frequency of the GNSS (1.5742 GHz for L1 *GPS*). This action increases the noise and, thus, decreases the signal-to-noise ratio (SNR) up to a critical point where the GNSS receiver cannot calculate its self-position. Another form of attack, called spoofing, aims to deceive the receiver into reporting a different, incorrect position by emitting modified, almost identical, GNSS signals. Therefore, addressing *GNSS* jamming and spoofing threats is a frequent concern in the realm of cyber warfare.

**Figure 1:**
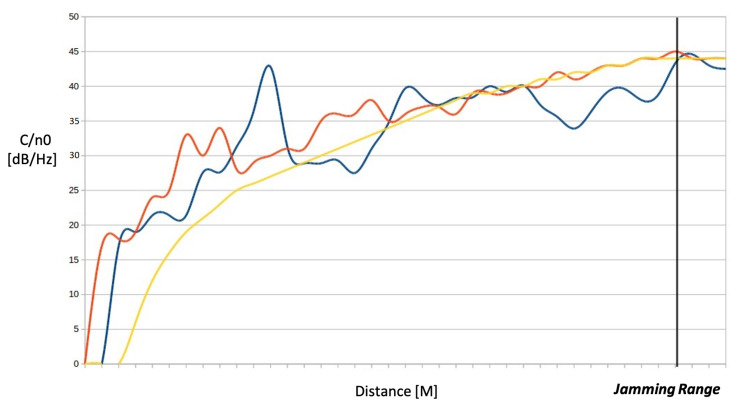
Jamming influence graph. As the distance between the jammer and the receiver increases, the receiver’s maximal SNR increases as well. The yellow, red, and blue lines represent theoretic behavior, external antenna recording, and smart-phone recording, respectively.

**Figure 2:**
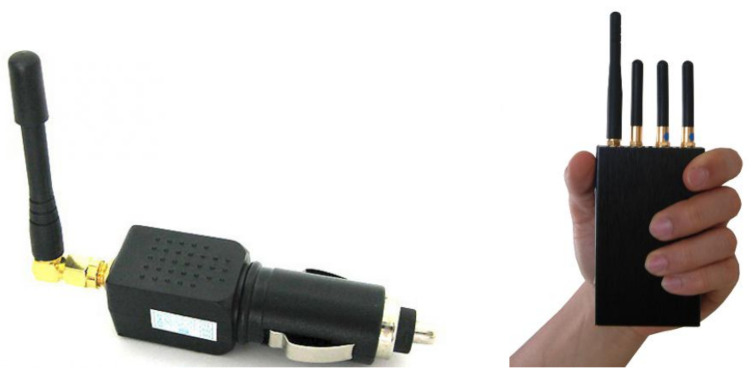
Commercial GNSS jammers: on (**left**), a jammer suited for vehicles; on (**right**), a more expensive portable GNSS/Wi-Fi/cellular jammer. The destructive potential of this jammer is massive.

A graph that demonstrates the effect a GNSS jammer on receivers is depicted in Figure 1, and a picture of cheap commercial *GNSS* jammers is depicted in Figure 2. The jamming range in Figure 1 is the maximum range for which the jammer can affect a GPS receiver. Note this this range is a direct function of the jammer strength.

The *x* axis in Figure 1 has no units and should be scaled according to the different transmitting powers. There are some methods to overcome the jamming problem. Multi-frequency receivers (L1, L2) work to some extent; however, they cannot cope with wide-band jammers. These days, most receivers are multi-constellation receivers (the American GPS, the Russian GLONASS, the Chinese BeiDou, and the European GALILEO). Since different GNSSs work in different frequencies, this can also work to some extent; however, it is not efficient in the case of intentional high-powered jammers. Assisted GNSS is very good but not accurate enough—a 30-m error is beyond the capacity of urban navigation. Differential GNSS is also very good but uncommon in commercial receivers that usually work with only one reference receiver.

In this work, we present how to harness the power of crowd-sourcing and particle filters to identify (intentional and accidental) jamming attacks on our most common cellular GNSS receivers. In addition, we are able to alert other users on the network and even identify the location of the jammer and its power. We suggest a probabilistic method for jamming detection and localization. Crowd-sourcing from several receivers and sensors enables jamming detection and localization.

### 1.1. Previous Works

In the scope of commercial GNSS receivers (smartphones), anti-jamming algorithms mainly focus on reducing the receiver’s sensitivity. Moreover, there is no systematic solution to this problem. In fact, automotive manufacturers start to look at more conventional approaches (dead reckoning) to face this issue. Utilizing crowdsourcing techniques to overcome jamming attacks was introduced by Scott [8]. The author suggested incorporating GPS jam-to-noise ratio detectors in cellphones to provide timely interference detection. Another work tried to address the jamming threat by utilizing smartphones as receivers [9]. However, the authors stated that jamming detection could not be achieved using smartphones in dynamic scenarios because those receivers rely solely on the receiver’s signal strength (C/N0) as an indicator. Their work confirmed that C/N0-based Android application detectors could work well in static scenarios. The authors also mentioned that this method is not suitable in dynamic scenarios because it cannot distinguish between decreased GPS signal strength and increased interference. Another approach for detecting GNSS signal jamming, when the direction of arrival is lost involves using the Fast Orthogonal Search (FOS) on the GPS data [10]. In this work the FOS method successfully detected the number of jammers simulated and their corrupted DOA signals. To date, Waze, Inc. has provided the best work for GNSS crowd-sourcing. The strength (and market value) of this company emerged from its vast sensor availability. Given that Waze can now easily detect traffic jams and road anomalies, it is plausible that using the already available GNSS data produced in every smartphone can provide new insights on susceptible jamming interference. The accuracy measure of the device, in turn, can be furthered utilized (e.g., to rely more on GNSS where GPS is interred, and vice versa). With the emergence of new vehicle-to-vehicle and vehicle-to-infrastructure technologies, many researchers have published works on the power of collaborative GNSS information and pseudoranges, along with additional in-car sensors and map-matching techniques to increase the accuracy, robustness, and reliability of the positioning information. The most common approach to fusion location information was Kalman filtering [11,12,13,14], extended Kalman filtering [15,16,17], cubature Kalman fileting [18], and particle filters [19,20,21]. Few studies have been conducted on implementing collaborative GNSS information to detect, alarm, or mitigate jamming or spoofing attacks for transportation applications, receivers against interference. One interesting work used cooperative adaptive cruise control to share raw GNSS information among cars driving in a convoy [22]. That work addressed a few assumptions on the current speed, distance, and pseudoranges transmitted from the lead vehicle to the following vehicles. An extensive overview of approaches for protecting GNSS can be found here [23]. Additional research worked toward integrating 802.11p dedicated short-range communication among vehicles sharing inertial measurement unit information with RF time-of-arrival measurements to supply some localization in GNSS-denied environments [16].

### 1.2. Our Contribution

The novelty of the proposed system is twofold:

(1) The algorithms presented are the basis for a GNSS coverage map framework.
(2) The probabilistic approach allows coping well with complicated jamming scenarios such as moving jammers). Further, the system can handle scenarios with multiple jammers. The proposed algorithm assumes no prior knowledge of the jammer’s transmitting strength; this information is computed during the Bayesian process. Both simulation and field experiment showed promising results.

## 2. Problem of Interest

This paper addresses two kinds of problems. The first problem, detecting whether jamming interference exist at a specific time-stamp, can be viewed as a case study of the more general systematic problem. Nevertheless, that case-study is extremely important for urban vehicle navigation. Knowing that the GNSS estimated position is not reliable (due to jamming interference), can be of great value for any navigation software (e.g., Waze).

**Figure 3:**
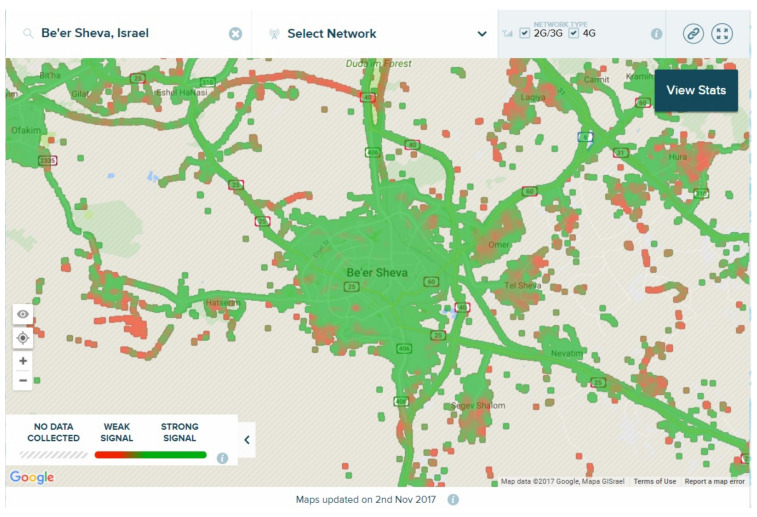
A cellular (2G/3G/LTE) coverage map of a city in Israel. One can clearly see the weak (red) zones in every street.

The second problem is to obtain a reliable GNSS coverage of a city. A great analogy can be given from cellular coverage maps. A London based company named OpenSignal has developed a cellular coverage map app. Thus, in every city in the world, one can see the coverage (reception) map of each available network provider. A typical coverage map is depicted in Figure 3.

The coverage map in Figure 3 is indifferent to time; that is, the coverage quality does not change from day to night. This is because the foremost factor that affects cellular coverage is the position of the antennas, a stationary feature by nature. The GNSS coverage or jamming pattern changes over time. Therefore, we suggest a slightly different model. The algorithm proposed in this paper can produce GNSS coverage maps that change in the course of the day. Such maps can aid authorities to gain insight regarding regions that may need a specific treatment. The well-known story of the truck driver who shut down the London Stock Exchange by installing a cheap GPS jammer in his vehicle [24] demonstrates both the importance and necessity of such a GNSS coverage map. Section 5 elaborates on the fabrication of a commercial off-the-shelf (COTS) internet-of-things device that can be installed in various spots and can gather the data necessary for a GNSS coverage map.

### Preliminaries

*A GNSS jammer* can be defined by its power, position and antenna patterns. The higher its power, the larger the radius of interference from its position. The antenna pattern suggests that not all jammers have an omni-pattern, and one can be in proximity to a jammer and sense no interference.

*A GNSS client* is a COTS sensor that records GNSS data. In the context of this paper, one can think of a client as a smartphone with an additional software layer. A moving vehicle is a good example of such a client.

*A jam↓* (**jam** with a down arrow) is an event when the client’s received SNR decreases. Such an event usually implies the client is approaching a jammer.

## 3. A Single Jammer Detection Algorithm

The following two sections explain the jamming algorithm. We begin by describing a simple version of the algorithm that can cope only with a single jammer. In the next section we present a more advanced version that can cope with multiple jamming scenarios. Both algorithms utilize particle filters in the convergence process. As opposed to the Kalman family, particle filters estimate the real distribution (posterior) by a number of parameters. For a detailed explanation regarding particle filters and their use within the context of navigation, we refer the reader to Thrun, Burgard, and Fox [25].

Almost all localization algorithms that utilize particle filters and treat each particle as a plausible position of the object they wish to follow. However, our scenario is comprised of two things–a client equipped with a GNSS device and unknown jammer(s). Thus, particle $x{\left(t\right)}^{\left[L\right]}$ is a candidate location for a jammer device at time *t*. Each particle holds a position, velocity (velocity=0 for stationary jammers), orientation, and transmitting power.

The proposed algorithm addresses the complicated jamming scenario problems:

- A moving jammer
- A dynamic transmitting power.

**Observation 1:** Moving jammers affect different clients in different ways. Assuming a jammer moves from *A* to *B*, then clients next to be *B* will report a jam↓ while clients next to *A* report the opposite.

**Observation 2:** Changes in the transmitting power affect all clients the same way. For example, assuming a jammer is turned off, then the received SNR will increase or hold for all clients at the same time.

Equipped with those observations, the algorithm spreads particles (jammers) within the range of interest (ROI), and each particle holds a random position, velocity, and transmitting power. Within each iteration, the particles move according to their initial velocity vectors. Then, each particle is evaluated according to its compatibility with the clients’ received SNR. Assuming a jammer exists in the client’s position, one can compute the interference on each client and compare it with the actual sensed interference; the tighter the matching, the higher the particle’s weight. The most important phase is re-sampling, wherein all the particles are re-sampled (with repetition) according to their proportional weight. Since the algorithm favors high weight particles, they are more likely to be duplicated.

However, particle filters do not supply a final answer, just a distribution in the form of *M* particles. One can define a convergence as the phase in which the distance between the center of mass and another particle does not exceed a predetermined threshold. Thus, in case of no jamming interference, the algorithm will not converge.

### The Uni-Modal Nature of the Particle Filter

**Figure 4:**
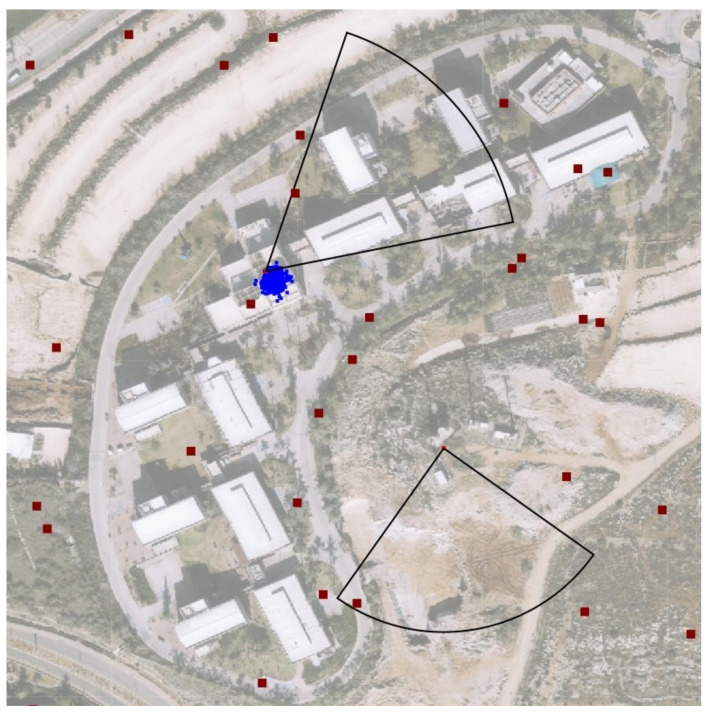
The uni-modal nature of a particle filter. Although two jammers operate within the ROI, the algorithm converges to only one of them.

A famous characteristic of the particle filter is it lack of bound to uni-modal (e.g., Gaussian) estimations [26]. In fact, because those filter are nonparametric, they can describe any probability density function. The multi-modal estimation does not assume a best guess but very wellcan handle two (or more) such guesses. Unfortunately, the very nature of this filter that allows for its multi-modal estimation ultimately “forces” the particle to converge to a single solution due to the re-sampling process. Each weighted re-sampling will eventually converge to a single value. In the literature, this problem is addressed as the kidnapping robot problem [25]. Figure 4 demonstrates this phenomenon. Two jammers operate within the ROI. Nevertheless, the algorithm converges only to one jammer. As the famous joke states, “It’s not a bug; it’s a feature of the inherent nature of the particle filter re-sampling process”.

The kidnapping robot problem is a scenario in which all the particles converge to the robot’s true location and then a foe (or a kid) “kidnaps” the robot and places it in unknown place. If the particles are not intelligently spread, the robot will not be able to re-locate itself. Although the solution to this problem is to re-spread a fraction (≈10) of the particles, it cannot be implemented in inherent multi-modal scenarios such as two (or more) jammers. In those scenarios, it is important to keep track of *n* different clusters of moving particles where *n*, the number of different jammers in the ROI is unknown. To address this issue, we modified the well-known K-Mean clustering algorithm. The K-Means demands *k*–the number of different clusters–as an input. This information is obscure in the proposed problem in two. We seek an algorithm that copes well with alternating jammers (partly time on, partly time off). The next section is dedicated to this problem.

## 4. Jammer Clustering Algorithm

This section presents a modified version of the previous single jammer detection algorithm. The main difference is its use of an additional clustering algorithm to both detect and track several jammers within the ROI. The subject of multi-target tracking (MTT) is considered a subproblem in the field of image processing and estimation [27]. Furthermore, approaching an MTT challenge by particle filter techniques has been suggested by [28,29].

As stated above, although particle filters are considered multi-modal filters (in the sense that they do not force a normal distribution), the innate nature of the re-sampling process tends to converges to a single solution. Therefore, the proposed clustering algorithm tackle this point. First, *M* particles are spread within the ROI, each with a position vector, velocity vector, and transmitting power. Once a convergence occurs, the algorithm **re-spreads** other *M* particles outside the region of interference of the first detected jammer. This ensures that the first jammer will not attract the new particles. The process of spreading, convergence, and re-spreading can be repeated several times. As opposed to the traditional K-Mean clustering, in which the number of clusters, k, must be known, the current algorithm assumes nothing regarding the real number of jammers in the scenario. A formal description of the clustering algorithm is given in Algorithm 1. The algorithm’s input is only the raw client sensor data (PVT and SNR) and the ROI. No assumptions are made.
**Algorithm 1:** A Clustering Jamming Algorithm 
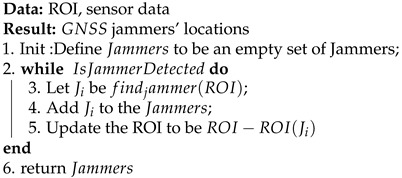


Algorithm 1 uses the single-jammer algorithm described in Section 3. Assuming the single-jammer algorithm converges, the function IsJammerDetected in Line 2 returns true. The heart of the algorithm lies in Lines 3–5. If a jammer was found, it will be added to the list. After that, the algorithm re-spreads particles **outside** the region of interference of the detected jammer.

### 4.1. Results

**Figure 5:**
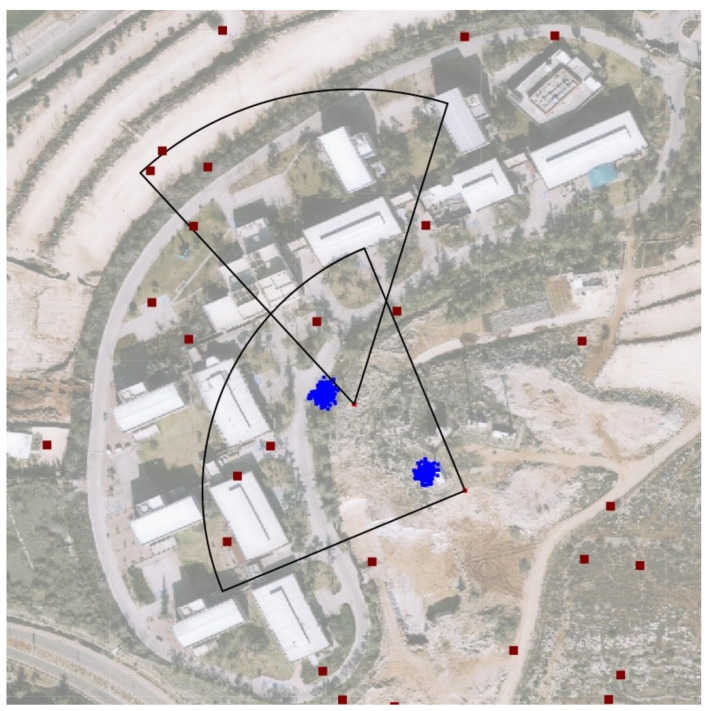
Several jammers localization and tracking. Even though the interference regions overlap, each set of particle tracks a separate jammer.

Figure 5 shows the output of the algorithm. In the figure, two jammers operate simultaneously. One can see from the figure that the jamming pattern in not omni but directed. The red dots in the figure represents clients (or smartphones). Furthermore, the jammers’ interference regions partly overlap. Nevertheless, the algorithm can converge two sets of *M* particles to both jammers. Thus, the algorithm both detects and tracks several jammers while tracing each jammer separately.

### 4.2. Computational Complexity

A well know fact is that unlike the Kalman Filter, a particle filter’s time complexity (the number of particles) scales exponentially [25]. Some works have been conducted to reduce the number of particles [30,31]. One must remember that each particle can be computed independently of the other, thus, the time complexity is O(n), where *n* is the number of particles. Doubling N will double the expected run-time. Alas, the configuration space grows exponentially with respect to the state vector’s size. As such, it is not recommended to extend a 2D particle filter to 3D.

## 5. Implementing a Dedicated Client

Since the total amount of the gathered data is rather small, any 32-bit micro-controller (e.g., ARM Cortex M4) can run the algorithm. The implication is that one of the sensor units can serve as the server. Moreover, the sensor unit size easily can be reduced to the size of a pack of cigarettes (including battery). On the other hand, the algorithm is flexible enough to run even on smartphones although only at 1 HZ sampling rate. The units themselves can operate in 10 Hz–an almost mandatory feature to detect dynamic jammers. Moreover, the Ublox 8-M GNSS receiver module that exists in most modern vehicles is supplied with a sensitive anti-jamming indicator. In fact, that indicator senses a continuous wave (CW) interference long before a smartphone GNSS module will.

## 6. Discussion

Previous works [28,29] have also suggested particle filters based methods but shown simulated results only. Mobile devices like smartphones hold little memory and cannot run heavy programming like simulators usually required. Herein, we have performed a field experiment in urban conditions that proves the method’s efficiency shown both in the results and in the rapid processing of the algorithm on a 32-bit micro-controller at 1 HZ sampling rate.

## 7. Jammer Implementation Using COTS Software Defined Radio (SDR) Hardware

The GNSS jammers are devices that “inject” noise in L1. In order to achieve this, we use the Blade-RF software defined radio (SDR) hardware. The noise (2 MHz bandwidth) was created using the GNURadio platform. The relative ease with which one can interrupt L1 GNSS signals is intolerable. One can easily detect jamming interference by inspecting the noise floor even when the smartphone is C/N0.

To efficiently implement a power-adjustable jammer, we decided to use a SDR COTS platform. This section describes the hardware and software requirements along with simple GNU Radio implementation and graphical use interfaces (GUI) followed by a short description of the experiment we conducted. As previously described, a jammer can be implemented by countless types of signals transmitted on the same frequency and with higher amplitude then the received signal strength [32]. For our experiment we implemented a CW and white noise signals at either L1 or L2 frequencies. The jamming signal has to be band-limited for both to be able to concentrate its power in the band of our choice and not interfere with other applications transmitting in close frequencies. In addition we wanted to be able to control and adjust the jamming power in order to contain the jamming radius and not disturb any GNSS based application, such as nearby base stations deriving their accurate timing from GNSS signals. We chose two SDR platforms: Nuand BladeRF and HackRF One. Both platforms not only answer our conditions, but also are easily programmed using free open-source tools such as GNU Radio. As opposed to similar works [33,34] that chose the Matlab Simulink environment, we wanted to demonstrate the ease of implementing such an experiment using open-source software [35].

The CW signal was used to estimate the transmitted power and its effect on the radius of the area being jammed. A transmitted power of about +10 dBm at CW helped us estimate the power spectral density of the noise we could generate in 1.5 MHz BW to be about 62 dB lower resulting about Pn = −52 dBm/Hz @ L1 = 1.575 GHz. This left us enough margin to account for any losses on our non-ideal antenna and allowed us to pass the noise power measurement using expensive test equipment. The Friis Free Space Loss model is defined in the following equation:

(1) $$L_{free-space}\left[dB\right]=20log\left(d\right)+20log\left(f\right)+20log\left(\frac{4\pi }{c}\right)$$

For L1=1575.42 Mz, f is given in Mhz and d is given in *m*, Equation (1) can be written as:

(2) $$L_{free-space}\left[dB\right]=20log\left(d\right)+36.38_{\left[dB\right]}$$

## 8. Conclusions and Future Work

Jamming interference is a big deal in the world of modern GNSS navigation. This paper presented a novel framework for GNSS jamming detection in different scenarios (multiple jammers, different antenna patterns). The Bayesian algorithm can provide solutions both for stationary and moving jammers scenarios. The experiments clearly show the the viability of the proposed solution. Moreover, the algorithm converges even in challenging condition where only 3 receiver are being used. Another important concept that was introduced in the paper was the “GNSS coverage map” (Section 2). We strongly believe that such a map can help spot potential (intentional and accidental) jammers within a city.

## Data Availability

Not applicable.
